# Immune tolerance induced by hematopoietic stem cell infusion after HLA identical sibling kidney transplantation

**DOI:** 10.3389/fimmu.2022.995243

**Published:** 2022-08-23

**Authors:** Hongfeng Huang, Qixia Shen, Jingyi Zhou, Xiuyan Yang, Qiuqin Cai, Jia Shen, Shi Feng, Wenqing Xie, Hong Jiang, Jianghua Chen

**Affiliations:** ^1^ Kidney Disease Center, The First Affiliated Hospital, College of Medicine, Zhejiang University School of Medicine, Hangzhou, China; ^2^ Key Laboratory of Nephropathy, The First Affiliated Hospital, College of Medicine, Zhejiang University, Hangzhou, China; ^3^ Institute of Nephropathy, Zhejiang University, Hangzhou, China

**Keywords:** hematopoietic stem cell infusion, immune tolerance, immunosuppression withdrawal, chimerism, GVHD, kidney transplantation

## Abstract

After the first attempt to induce operational tolerance, it has taken decades to implement it in clinical practice. Recipients with Human leukocyte antigen (HLA) identical sibling donors were enrolled. Hematopoietic stem cells (HSCs) infusion was done after HLA identical sibling kidney transplantation (KTx). Three cases included were followed up for over 8 years. The perioperative conditioning protocol included anti-CD20, rabbit anti-thymocyte globulin (ATG), total lymphoid irradiation (TLI), and cyclophosphamide. Infusion of CD3^+^ cells and CD34^+^ cells was conducted. The withdrawal of immunosuppression was determined by mixed lymphocyte reaction (MLR) and graft biopsy. Case 1 and Case 2 showed persistent chimerism, while chimerism was not detected in Case 3. All three recipients showed a low-level response to donor-specific stimulation. Case 1 and Case 3 met the withdrawal rules at 16 and 32 months after transplantation, respectively. Graft function was stable, and no rejection signs were observed in routine biopsies until 94 and 61 months after transplantation. Case 2 was diagnosed with graft-versus-host disease (GVHD) 9 months after transplantation and recovered after an enhanced immunosuppression therapy. Steroids were withdrawn after 1 year, and 0.5 mg tacrolimus twice a day is currently the only immunosuppression at 8 years and 8 months. In conclusion, our clinical experience indicated the efficacy of non-myeloablative conditioning protocol for tolerance induction in HLA identical patients. Complete chimerism might be a risk factor for GVHD.

## Introduction

The short-term survival of kidney transplants has improved significantly due to the development of highly efficacious immunosuppressive agents (1). In the United States, the average 1-year and 5-year graft survival of deceased-donor kidney transplant recipients is 94.3% and 76.3%, respectively. For living-donor kidney transplant recipients, the average 1-year and 5-year graft survival is 97.8% and 86.5%, respectively (2). Long-term outcomes remain unsatisfactory because of the current need for lifelong immunosuppression, which may increase the risks of cardiovascular disease and malignancies. Chronic rejection remains the leading cause of long-term graft loss. Immune tolerance inducement after transplantation is the ultimate solution to these problems.

After the first attempt to induce operational tolerance (3), it has taken decades to implement it in clinical practice. Among all kinds of theories, mixed chimerism induced by bone marrow/hematopoietic cell transplantation is best acknowledged. Mixed chimerism is defined as a state in which donor and recipient hematopoietic cells coexist (4). Three centers in the United States have conducted clinical trials for inducing recipient chimerism-based kidney transplantation tolerance (5). Massachusetts General Hospital took four out of 19 recipients off immunosuppression under the protocol of spontaneous kidney and bone marrow transplantation with non-myeloablative conditioning, and an 8–14-month course of calcineurin inhibitor with or without rituximab (6). Stanford researchers succeeded in taking 16 out of 38 HLA-matched and HLA-mismatched patients off immunosuppression by combining living-donor kidney and hematopoietic cell transplantation with posttransplant conditioning using total lymphoid irradiation and anti-thymocyte globulin (ATG) (7). Northwestern protocol achieved 50.00% and 63.15% immunosuppression withdrawal, respectively, in HLA-matched and HLA-mismatched groups with a novel approach using a bioengineered mobilized cellular product enriched for HSCs and tolerogenic graft facilitating cells combined with non-myeloablative conditioning (8). However, the sample sizes and results remain insufficient. Moreover, there is no evidence from Asian populations of immune tolerance induced by HSCs after kidney transplantation.

Herein, we shared our clinical experience of three HLA identical sibling kidney transplantation patients with HSC infusion and other conditioning who were followed up for 8–9 years.

## Materials and methods

### Patients and ethics

**Figure 1:**
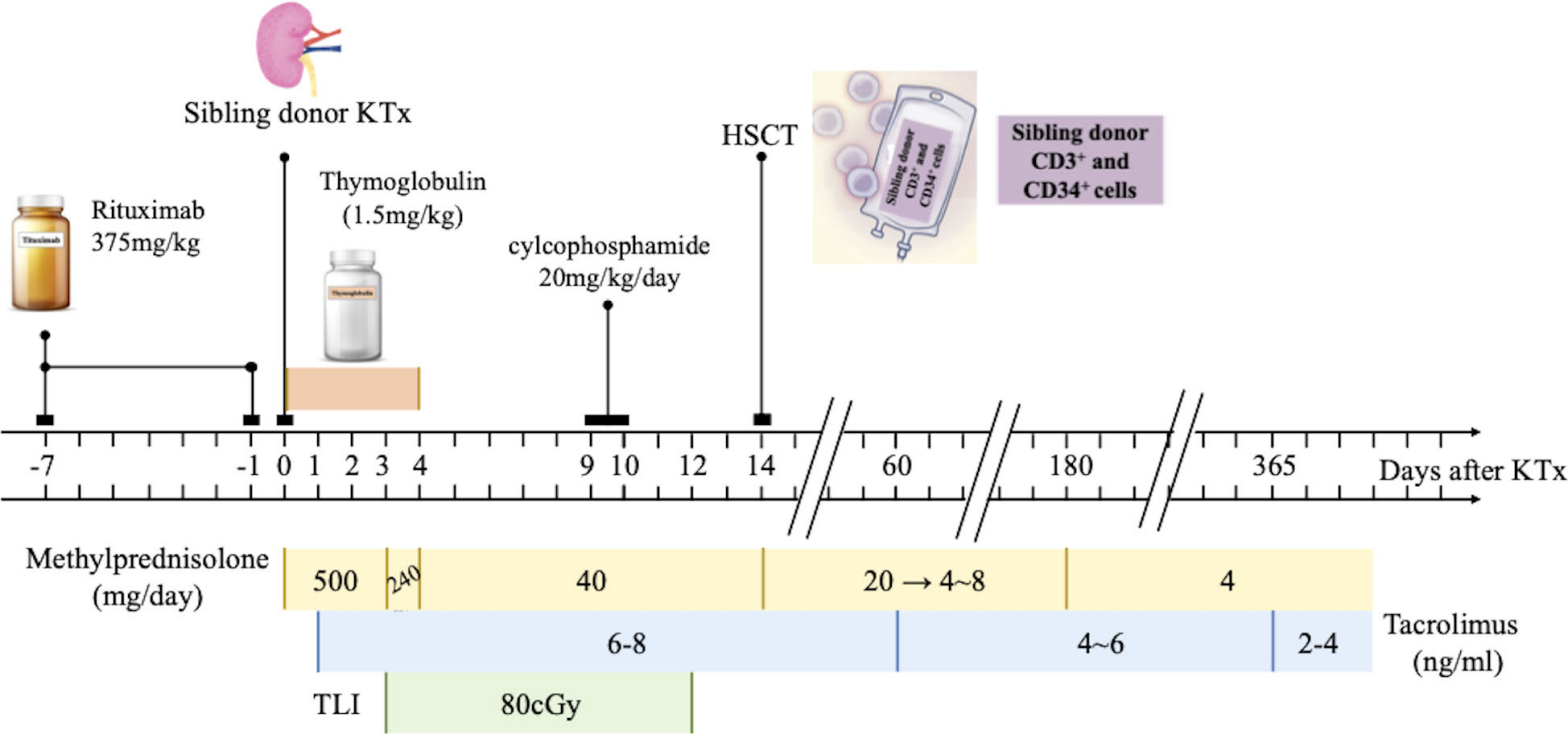
Kidney transplantation tolerance induction by sequential CD20 B cell-deleted, T Cell-deleted and sibling HSCT in Three Patients with end-stage renal disease (ESRD). Three patients with (ESRD) received a tolerance induction regimen consisting of rituximab, an ti- thymocyte globulin, total lymphoid irradiation, cyclophosphamide and hematopoietic stem-cell transplantation (HSCT). The kidney grafts were obtained from their siblings. Methylprednisolone (500 mg from Day 0 to Day 2,240 mg on Day 3, 40 mg from Day 4 to D ay 14) intravenously administered. Oral dose of 20 mg was given from Day 15 and reduced by 4 mg/day to 8 mg/day maintenance dose until six months after operation. After si x months, the maintenance dose was 4 mg/day. The target trough level concentration within two months was 6-8 ng/ml, within I year was 4-6 ng/ml and after 1 year was 2-4 ng/ml. KTx, kidney transplantation; ESRD, end-stage renal disease; HSCT, hematopoietic stem cell transplantation.

Three recipients with end-stage renal disease (ESRD) were enrolled. They had HLA identical sibling donors. The research protocols were registered in the Chinese Clinical Trials Registry (ChiCTR-ONC-11001873) and approved by the Ethics Committee of the First Affiliated Hospital, Zhejiang University [Subject 1: 2008-YJ6-02 (108); Subject 2: 2009-YJ6-01(024); Subject 3: 2009-YJ6-01(082)]. Both donors and recipients signed consent forms before inclusion. The detailed protocol is shown in **Figure 1**.

### Perioperative recipient preconditioning

Anti-CD20 (rituximab, Roche) was given intravenously on Day -7 and Day -1 before transplantation (375 mg/kg per dose). Rabbit ATG (Thymoglobulin, Genzyme) was administered from Day 0 to Day 4 (1.5 mg/kg per dose). Recipients received 10 doses per day of 80 cGy TLI from Day 3 (superior and inferior phrenic lymph nodes, thymus, spleen, and para-aortic lymph node). Cyclophosphamide was given on Day 9 and Day 10 (20 mg/kg per dose).

### Immunosuppressive therapy

Three doses of 500 mg methylprednisolone were intravenously administered from Day 0 to Day 2. The dose was decreased to 240 mg on Day 3. From Day 4 to Day 14, it was further tapered to 40 mg. An oral dose of 20 mg was given from Day 15 and was reduced by 4 mg/day to 8 mg/day maintenance dose until 6 months after the operation. After 6 months, the maintenance dose was 4 mg/day. Tacrolimus was given at an initial dose of 0.1 mg/kg from Day 1. The target trough level concentration within 2 months was 6–8 ng/ml, within 1 year was 4–6 ng/ml, and after 1 year was 2–4 ng/ml.

### Collection of donor HSCs

Donors received a 5-day course of granulocyte colony-stimulating factor (G-CSF) from Day 9 at the dose of 6 μg/kg per day (filgrastim, Kyowa Hakko Kirin China Pharmaceutical). Mononuclear cells were collected on Day 13 and Day 14 by apheresis (CS-3000, Baxter) with anti-CD34 monoclonal antibody (BD Company). The numbers of CD3^+^ cells and CD34^+^ cells were calculated by flow cytometry, and the cells were infused into recipients on Day 14 (dosage in **Table 1**).

**Table 1:** Mononuclear cell infusion dose and follow-up information.

Case	CD34^+^ cell dose (×10^6^ kg^-1^)	CD3^+^ cell dose (×10^6^ kg^-1^)	Serum creatinine at last visit (μmol/L)	Duration of chimerism (months)	Duration off drugs (months)
1	3.7	70	89	12	91
2	6.0	110	130	90	0
3	1.5	25	104	0	60

### Chimerism

The time points for serial chimerism measurements were 1, 3, 6, 12, 24, 36, 60, and 96 months after infusion. The percentage of donor type cells was determined by short tandem repeats (STR)-PCR (9, 10) (ABI9700, PowerPlexFusionSystem). Complete chimerism is >95% of donor-type cells, mixed chimerism is 2.5%–95% of donor-type cells, and microchimerism is <2.5% of donor-type cells (11, 12).

### Lymphoid cell stimulation

Lymphoid cells of donors and recipients were separately isolated and then concentrated to 1 × 10^6^/ml. Donor lymphoid cells were cultured with mitomycin C, and recipient lymphoid cells were labeled with Carboxyfluorescein succinimidyl ester (CFSE) (Thermo C1157) dye. Recipient lymphoid cells alone were used as the control group. Mixed culture of both donor and recipient cells was used as the stimulation group. After 3 days at 37°C in the culture chamber, fluorescence intensity and cell proliferation were detected. Stimulation index (SI) was defined as the ratio of fluorescence intensity between the stimulation group and the control group.

### Graft biopsy

Routine allograft biopsies were taken on Day 0, 1 month before immunosuppression withdrawal, and 1 year and over 4 years after immunosuppression withdrawal. Indication biopsies were taken during any episode of unexplained graft dysfunction. Diagnosis was made according to Banff 05.

### Immunosuppression withdrawal indications

Indications for immunosuppression withdrawal are as follows: no episodes of rejection and GVHD, serum creatinine fluctuation within 20%, at least three times of 24-h urine protein <0.3 g within 6 months, no evidence of rejection by biopsy, and MLR showed a low-level response to donor-specific stimulation. Steroids were withdrawn when all of the conditions were met. Tacrolimus was stopped after 1 month of use at a concentration of 2–3 ng/ml.

## Results

### Patients’ baseline characteristics

The three recipients were all ESRD patients with no biopsy records for primary diagnosis before the operation. The median duration of hemodialysis was 5 months. panel reactive antibodies (PRAs) were all negative (Luminex). Flow cytometry crossmatch was also negative. Detailed information is shown in **Table 2**.

**Table 2:** Patients’ baseline characteristics.

	Case 1	Case 2	Case 3
**Recipient age (year)/gender**	38/F	34/M	55/M
**Donor age (year)/gender**	42/F	53/F	58/F
**Relationship**	Sisters	Brothers	Siblings
**PRA**	Negative	Negative	Negative
**Crossmatch**	Negative	Negative	Negative
**HLA mismatch**	0	0	0
**Hemodialysis duration**	8 months	2 months	6 months
**Blood type**	O-O	O-O	A-A
**Underlying diseases**	No	No	No
**Drugs for long-term use**	No	No	No

PRA, panel reactive antibody; HLA, human leukocyte antigen.

### Conditioning and immunosuppression withdrawal

Case 1 and Case 3 met the withdrawal rules at 16 and 32 months after transplantation, respectively. Steroids were weaned first, and the trough concentration of tacrolimus was reduced to 2–3 ng/ml until withdrawal after 1 month. Graft function was stable, and no rejection signs were found in routine biopsy after 94 and 61 months of follow-up. Case 2 suffered from itchy hypoallergenic rashes on the back and forearms at Month 9, and skin biopsy showed chronic mucosal inflammation. Meanwhile, bilirubin and enzymes of the liver, including alanine amino transferase (ALT) and aspartate amino transferase (AST), increased slightly, serum creatinine ascended from 101 to 165 μmol/L, and urine protein was 0.624 g/24 h. Indicated graft biopsy showed acute tubular injury with glomerular sclerosis and glomerular mild changes (**Table 3**). Considering the constantly >95% chimerism rate, GVHD I was diagnosed. An enhanced immunosuppression therapy with 40 mg/day of intravenous methylprednisolone was administered for 7 days. The patient recovered after 14 days. His rash subsided, the liver function was recovered, and creatinine dropped to 106 μmol/L and stabilized around 100 μmol/L. Steroids were withdrawn after 1 year. Currently, 0.5 mg tacrolimus twice a day is the only immunosuppression given, with stable graft function (follow-up duration of 8 years and 8 months).

**Table 3:** Routine biopsy of kidney grafts.

Case	Day 0	1 month before withdrawal	Year 2	Over Year 4
1	Kidney tissue with glomerular sclerosis (1/16)	Normal graft (Month 16)	Normal graft (12 months after withdrawal)	Kidney tissue with 4/37 glomerular sclerosis (94 months after KTx)
2	Kidney tissue with glomerular sclerosis (3); Mesangial tissue mild hyperplasia and IgA deposition	Acute tubular injury with glomerular sclerosis; Glomerular mild changes (Month 9 with GVHD)		Normal graft (51 months after KTx)
3	Kidney tissue with glomerular sclerosis (1/28)	Normal graft (Month 32)		Normal graft (61 months after KTx)

KTx, kidney transplantation; GVHD, graft-versus-host disease.

### Graft function monitoring

**Figure 2:**
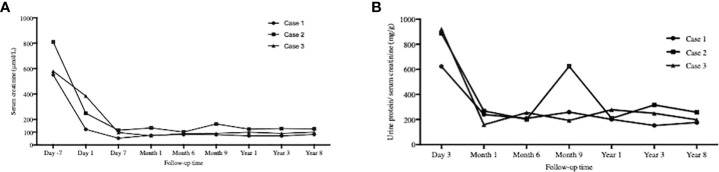
Graft function monitoring. The serial time points were 7days before KTx, day 1, day 7 and 1, 6, 9, 12, 36, 96 months after KTx for **(A)** Serum creatine mornitoring and day 3 and 1, 6. 9, 12, 36, 96 months after KTx for **(B)** Urine protein/serum creatinine. The graft function of Case 1 and Case 3 remained stable. Case 2 experienced a transient elevation in serum creatinine and urine protein when graft-versus-host disease occurred. KTx, kidney transplantation.

The graft function of Case 1 and Case 3 remained stable before and after medication withdrawal. Case 2 experienced a transient elevation in serum creatinine and urine protein when GVHD occurred. The monitoring of serum creatinine and urine protein/creatinine ratio is shown in **Figures 2A, B**, respectively.

### Chimerism monitoring

**Figure 3:**
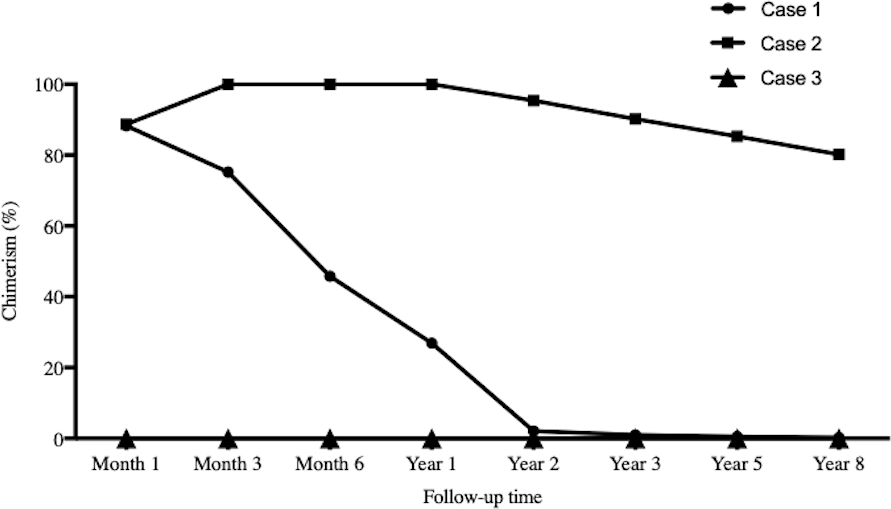
Chimerism monitoring. The time points for serial chimerism measurements were I , 3, 6, 12, 24, 36, 60 and 96 months after infusion. Case I and Case 2 showed chimerism rate >75%. Case I experienced gradual decrease in chimerism rate until maintaining micro-chimerism status from Month 24. Case 2 showed mixed chimerism in the early stage and met the standard of complete chimerism at Month 3. There was no chimerism status detected in Case 3.

As **Table 1** showed, the dose for Case 3 was much lower than those of the other two cases. Case 1 and Case 2 showed chimerism rate >75% (**Figure 3**). Case 1 experienced a gradual decrease in chimerism rate until maintaining micro-chimerism status from Month 24. Case 2 showed mixed chimerism in the early stage and met the standard of complete chimerism at Month 3. Thereafter, his chimerism rate reverted to mixed chimerism. There was no chimerism status detected in Case 3.

### Biopsy results

**Figure 4:**
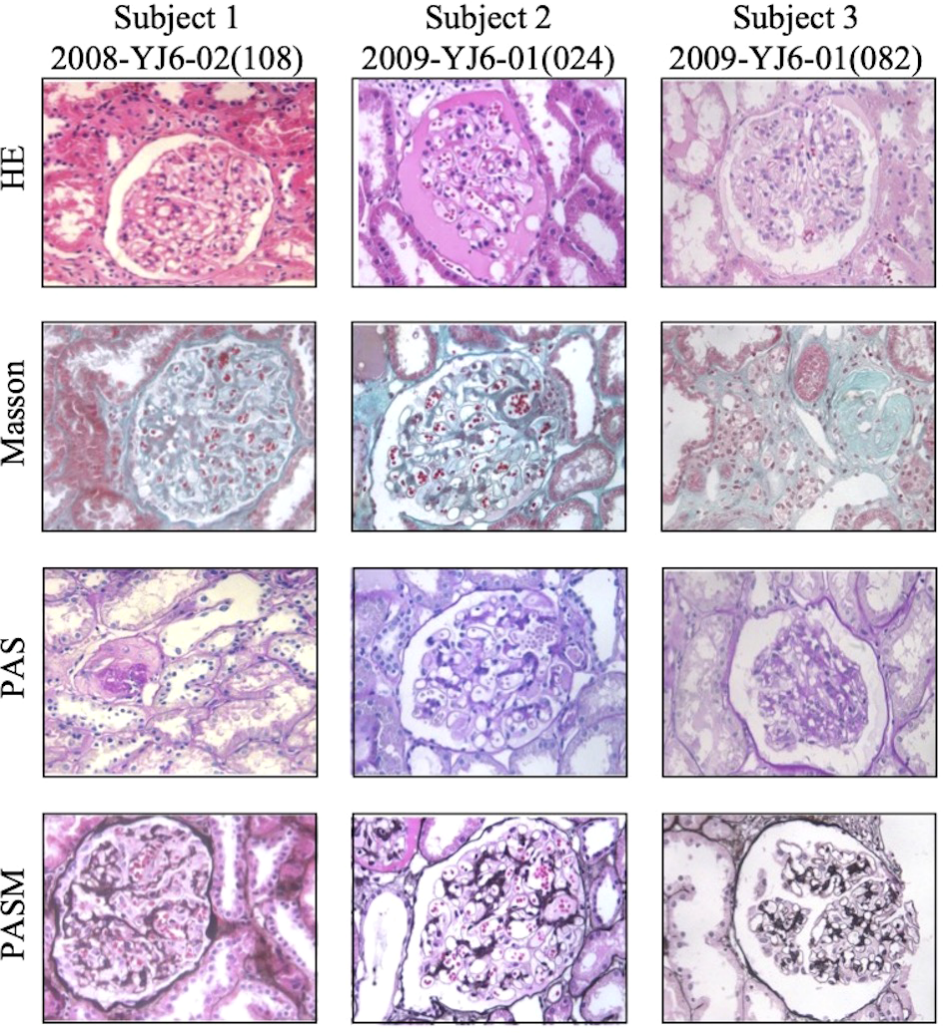
Representative pictures of tbe latest kidney allograft biopsy staining of three subjects. Tbe time of biopsy after KT above for subject I, subject 2 and subject 3 was 6 years, 5 years and 12 years, respectively. No signs of rejection was recognized. Pictures were taken at 400x magnification.

Case 1 and Case 3 showed ordinary graft pathology within the follow-up duration (**Table 3**). Case 2 suffered from GVHD and underwent indicated graft biopsy as mentioned above. The last time of kidney graft biopsy after KT for subject 1, subject 2, and subject 3 was 6 years, 5 years, and 12 years, respectively (**Figure 4**). No episode of rejection was recognized.

### SI

**Figure 5:**
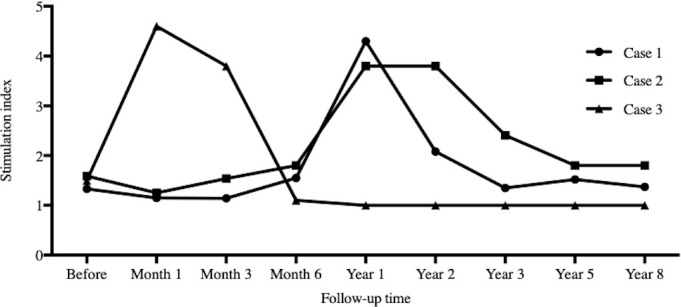
Stimulation index. The time points for serial stimulation index records were I , 3, 6, 12, 24, 36, 60 and 96 months after infusion. All three recipients showed low level of stimulation index during the follow-up.

All three recipients showed a low level of SI at the end of follow-up as shown in **Figure 5**, that is, host lymphocytes were tolerant to donors’ stimulation. Case 3 was the most obvious.

### Blood cell monitoring

**Figure 6:**
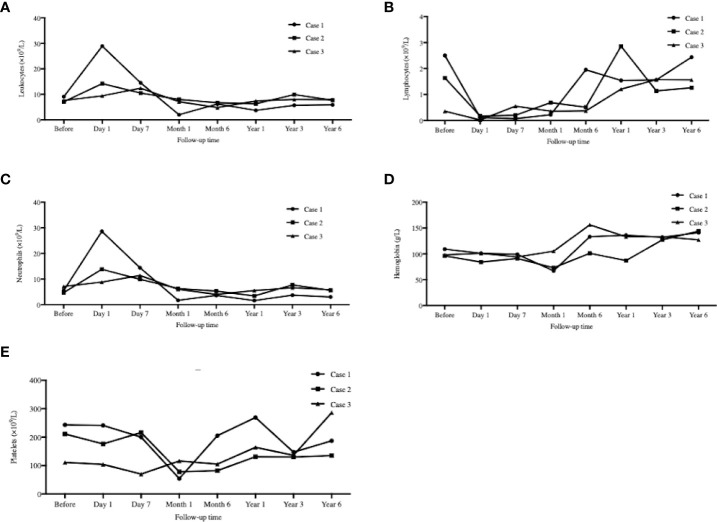
Peripheral blood cell counts mornitoring. The serial peripheral blood cell counts monitoring at day 1, day 7 and 1, 6, 12, 36, 72 months after transplantation. **(A)**: Leukocytes; **(B)** Lymphocytes; **(C)**: Neutrophils; **(D)**: Hemoglobin; **(E)**: Platelets. All types of blood cells decreased after transplantation (except ncutrophils), and then gradually recovered.

All types of blood cells decreased after transplantation (except neutrophils), and then gradually recovered (**Figures 6A–E**). The average turning points for neutrophils, lymphocytes, hemoglobin, and platelets were Week 3, Week 3, Weeks 2–4, and Month 1, respectively.

### Complications and adverse events

Donors suffered from transient G-CSF side effects, including dizziness and back pain. Case 1 was diagnosed as having pulmonary infection in 8 months after KTx and osteoporosis and premature ovarian failure in 9 months after KTx. Case 2 developed lung tuberculosis at Month 12 and recovered after 12-month efficient treatment. He also suffered from pulmonary aspergillosis at Month 16 and recovered after voriconazole anti-aspergillosis therapy. The other two patients had no pulmonary infection.

## Discussion

There are multiple definitions and explanations for immune tolerance in transplantation, of which graft function well after 1 year of immunosuppression withdrawal is a generally accepted one (13). It is easier for liver transplants (14–16), while kidney transplantation needs enhanced preconditioning to induce operational tolerance (1).

Three cases of HLA identical sibling kidney transplantation were enrolled in this study. Conditioning included rituximab, rabbit ATG, TLI, and cyclophosphamide. Donor peripheral HSCs were infused on Day 14. Case 1 and Case 3 were weaned off immunosuppression at Months 17 and 33, respectively. They had stable graft function and were considered to be immune tolerant. However, the mechanisms for their tolerance might be different. Case 1 had a significantly high rate of chimerism shortly after transplantation and maintained micro-chimerism later. This was in accordance with Prof. Strober’s opinion that chimerism rate >65% for >60 days was the indication of donor HSC successful residency and the host development of central tolerance (1). Case 3 did not show any evidence of chimerism, which might result from the lower dose of both CD34^+^ and CD3^+^ cell infusion. We had two hypotheses for his tolerance. One was that STR-PCR was not sufficiently sensitive for chimerism detection (1), as donor HSCs may reside in host bone marrow/thymus. The other was that conditioning could change host immune patterns, for example, Th1 to Th2 and Th17 to Treg (1, 17). The mixed lymphocyte culture and stimulation confirmed the existence of tolerance. Case 2 developed Mass General Hospital (GVHD). According to the report from MGH, it was due to the excessively high-level chimerism (18). Currently, Case 2 is on minimal tacrolimus maintenance along with stable graft function, implying his potential for immunosuppression withdrawal.

There is no consensus on the protocol for immunosuppression withdrawal among different centers. In Northwestern University’s experience, tacrolimus was replaced by sirolimus (trough levels of 8–10 ng/ml) 3 months posttransplantation. Mycophenolic acid was weaned at 12–18 months and sirolimus at 18–24 months on a clean renal biopsy premise. All complete immunosuppression withdrawal in this study might result from the cases only detecting transient chimerism (19). Monitoring clinical indications, including graft function and biopsy, was the main way to decide on withdrawal, the same as in Case 3 in our study. In contrast, Stanford University emphasized the importance of chimerism. Its standard for withdrawal was persistent chimerism for at least 6 months, no clinical rejection episodes, no GVHD, and no rejection signs on a protocol biopsy within 2 weeks before the discontinuation of immunosuppressive drugs (7).

The most critical problems are how to regulate the chimerism rate and the maintenance. As approved by Northwestern and Stanford universities, we theorize that the two key points are conditioning and cell infusion dose (5). The more powerful the conditioning is, the higher the chance of chimerism. The early Northwestern protocol only used two doses of alemtuzumab for conditioning and 3.74–14.40 × 10^6^/kg CD34^+^ cells for four-times infusions (19). All 17 patients detected transient micro-chimerism, and only seven succeeded in withdrawal. Later, researchers improved the conditioning (fludarabine, cyclophosphamide, and total body irradiation were added) and infusion (genetically modified facilitating cells were added) protocols (1, 20). Even for HLA-mismatched patients, they achieved 16 cases of chimerism out of 19 cases, and 12 of them stopped all immunosuppressive medications. Stanford protocol employed more CD3^+^ cells to help clonal deletion further and facilitate CD34^+^ cell residency. They succeeded in inducing chimerism in 21 out of 22 HLA-matched cases. Later, 17 of them were weaned off immunosuppression. Even in the 10 cases of HLA-mismatched transplantation, nine developed chimerism with increased CD3^+^ cell infusion. The experience from these two centers suggested that clonal deletion of host lymphocytes by either enhanced conditioning or CD3^+^ cell infusion could help chimerism induction. However, avoidance of complete chimerism is essential to reduce GVHD risk. Conditioning enhancement can also lead to high risks of infection. Our Case 2 was a typical example. We should pay concern to rejection after weaning off immunosuppression, which presented a rate of 13.8% during off drugs in three centers (1). Although it could be reversed, it may be followed by graft loss.

Our future study will focus on HLA-haplo-matched/mismatched cases to further explore an optimal protocol for kidney transplant immune tolerance induction and the underlying mechanisms.

## Data availability statement

The original contributions presented in the study are included in the article/Supplementary Material. Further inquiries can be directed to the corresponding authors.

## Ethics statement

The studies involving human participants were reviewed and approved by the Ethics Committee of the First Affiliated Hospital, Zhejiang University. The patients/participants provided their written informed consent to participate in this study. Written informed consent was obtained from the individual(s) for the publication of any potentially identifiable images or data included in this article.

## Author contributions

In this work, HH, JC and HJ conceived and designed the experiments. HH and QS drafted the manuscript. JZ, JS, XY, QC, SF and WX collected human samples. HH, JZ, and QS performed the experiments and data analysis. HH, QS, JZ, and HJ interpreted the results of the experiments. HH, QS, and HJ edited and revised the manuscript. HH, QS, and JZ prepared the figures. All authors approved the final version of the manuscript.

## Funding

This work was supported by grants from the Natural Science Foundation of Zhejiang Province, Zhejiang, China (Grant No. LY20H020003), Medicine and Health Science and Technology Plan Projects of Zhejiang Province, Zhejiang China (Grant No. 2019KY377) and the National Natural Science Foundation of China (NSFC 82070767, 81770750).

## Conflict of interest

The authors declare that the research was conducted in the absence of any commercial or financial relationships that could be construed as a potential conflict of interest.

## Publisher’s note

All claims expressed in this article are solely those of the authors and do not necessarily represent those of their affiliated organizations, or those of the publisher, the editors and the reviewers. Any product that may be evaluated in this article, or claim that may be made by its manufacturer, is not guaranteed or endorsed by the publisher.

## Abbreviations

HLA, human leukocyte antigen; HSCs, Hematopoietic stem cells; KTx, kidney transplantation; ATG, anti-thymocye globulin; TLI, total lymphoid irradiation; MLR, mixed lymphocyte reaction; GVHD, graft-versus-host disease; ESRD, end-stage renal disease; G-CSF, granulocyte colony-stimulation factor; SI, stimulation index.
