# Corrigendum: Immune tolerance induced by hematopoietic stem cell infusion after HLA identical sibling kidney transplantation

**DOI:** 10.3389/fimmu.2022.1052676

**Published:** 2022-10-19

**Authors:** Hongfeng Huang, Qixia Shen, Jingyi Zhou, Xiuyan Yang, Qiuqin Cai, Jia Shen, Shi Feng, Wenqing Xie, Hong Jiang, Jianghua Chen

**Affiliations:** ^1^ Kidney Disease Center, The First Affiliated Hospital, College of Medicine, Zhejiang University School of Medicine, Hangzhou, China; ^2^ Key Laboratory of Nephropathy, The First Affiliated Hospital, College of Medicine, Zhejiang University, Hangzhou, China; ^3^ Institute of Nephropathy, Zhejiang University, Hangzhou, China

**Keywords:** hematopoietic stem cell infusion, immune tolerance, immunosuppression withdrawal, chimerism, GVHD, kidney transplantation

In the published article, there was an error in the Funding statement. Previously, it read: “This work was supported by grants from the Natural Science Foundation of Zhejiang Province, Zhejiang, China (Grant No. LY20H020003) and Medicine and Health Science and Technology Plan Projects of Zhejiang Province, Zhejiang China (Grant No. 2019KY377).” However, two more fundings supporting this research, the National Natural Science Foundation of China (NSFC 82070767, 81770750), should also be added to the Funding section. The correct Funding statement appears below.

The authors apologize for this error and state that this does not change the scientific conclusions of the article in any way. The original article has been updated.

## Funding

This work was supported by grants from the Natural Science Foundation of Zhejiang Province, Zhejiang, China (Grant No. LY20H020003), Medicine and Health Science and Technology Plan Projects of Zhejiang Province, Zhejiang China (Grant No. 2019KY377) and the National Natural Science Foundation of China (NSFC 82070767, 81770750).

## Publisher’s note

All claims expressed in this article are solely those of the authors and do not necessarily represent those of their affiliated organizations, or those of the publisher, the editors and the reviewers. Any product that may be evaluated in this article, or claim that may be made by its manufacturer, is not guaranteed or endorsed by the publisher.

